# Cerebrospinal fluid biomarkers in cerebral amyloid angiopathy: insights from a clinical case series

**DOI:** 10.1055/s-0045-1812887

**Published:** 2025-11-28

**Authors:** Ana Silvia Sobreira Lima Verde, Alessandra Braga Cruz Guedes de Morais, Amanda Vale Catunda, João Igor Dantas Landim, Ian Silva Ribeiro, Bruno Diógenes Iepsen, Norberto Anízio Ferreira Frota

**Affiliations:** 1 Hospital Geral de Fortaleza, Departamento de Neurologia, Fortaleza CE, Brazil.; 2 Universidade Federal de São Paulo, São Paulo SP, Brazil.; 3 Universidade de Fortaleza, Fortaleza CE, Brazil

**Keywords:** Cerebral Amyloid Angiopathy, Biomarkers, Amyloid Beta-Peptides, Cerebrospinal Fluid

## Abstract

Cerebral amyloid angiopathy (CAA) is a small vessel disease characterized by the deposition of amyloid-beta in small cerebral vessels, which can lead to intracerebral hemorrhages and cognitive impairment. Rare variants, such as cerebral amyloid angiopathy-related inflammation (CAA-ri) and iatrogenic CAA (ICAA), may mimic other neurological conditions and challenge diagnosis in clinical practice. We present three cases that illustrate distinct CAA syndromes, along with CSF biomarker analysis. One patient experienced recurrent hemorrhagic strokes with a history of dural graft, raising concerns about amyloid transmission. Two patients presented with rapidly progressive dementia that fulfilled CAA-ri criteria. All cases exhibited decreased levels of CSF Aβ40 and Aβ42, with one showing elevated p-tau, suggesting comorbid Alzheimer's pathology. Cerebrospinal fluid biomarkers complement neuroimaging in the diagnosis of CAA, aiding in differentiation from other dementias. Early recognition and diagnosis of CAA-ri variants is crucial, because immunotherapy may improve outcomes. Further research is necessary to establish biomarker thresholds and their clinical applicability.

**TEACHING POINTS**

1. Cerebral amyloid angiopathy (CAA) can present with a broad clinical spectrum; therefore, recognition can be challenging, and the diagnostic criteria may lack accuracy, particularly in cerebral amyloid angiopathy-related inflammation (CAA-ri) variants.
2. Early recognition and diagnosis of CAA-ri variants allows for timely immunotherapy, potentially improving outcomes.
3. Cerebrospinal fluid (CSF) biomarkers (low Aβ40, Aβ42) may be useful adjuncts in diagnosing CAA and CAA-ri.

## CLINICAL VIGNETTE

### Case 1

**Figure 1:**
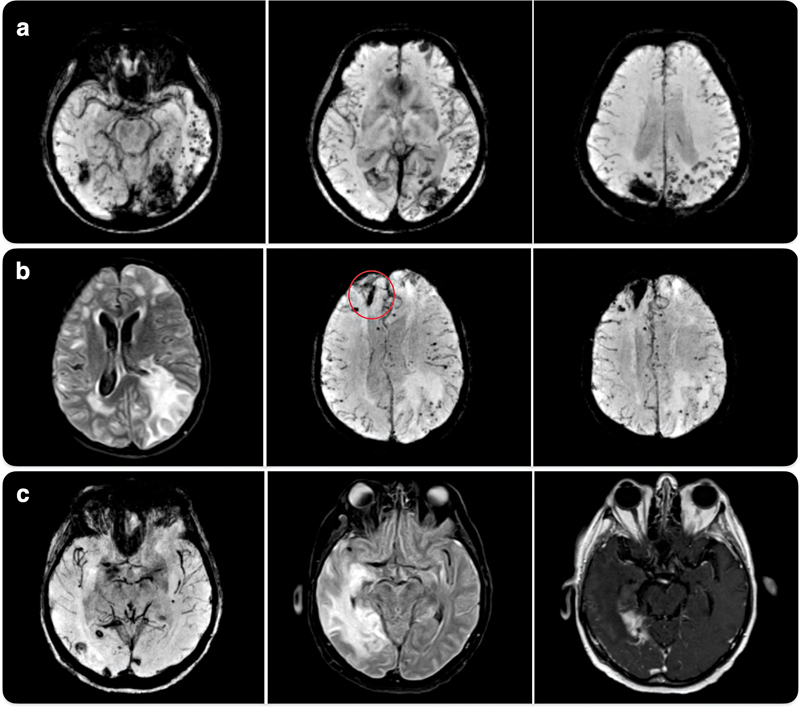
Brain magnetic resonance imaging (MRI) findings a. Case 1: Axial susceptibility-weighted imaging (SWI) MRI reveals multiple cortical and subcortical lobar microbleeds, predominantly in posterior regions, b. Case 2: Axial T2-weighted and SWI MRI demonstrate cortical and subcortical microbleeds, mainly in the left hemisphere, associated with asymmetric vasogenic edema c. Case 3: Axial SWI (left), fluid-attenuated inversion recovery (FLAIR) (center), and post-contrast T1-weighted (right) MRI show lobar microbleeds, cortical-subcortical vasogenic edema, and focal leptomeningeal enhancement, respectively.

A 45-year-old man presented with recurrent lobar hemorrhagic strokes beginning at 42 years old, affecting the bilateral occipitotemporal regions. He had a history of traumatic brain injury at 4 years old with dural graft placement. Brain magnetic resonance imaging (MRI) revealed multiple lobar microbleeds fulfilling Boston 2.0 criteria for probable CAA ( Figure 1 ). Cerebrospinal fluid biomarkers showed reduced Aβ40 and Aβ42 levels ( Table 1 ). Given the early onset, compatible imaging, biomarker profile, and prior neurosurgical exposure, the diagnosis of probable iatrogenic CAA was established. No immunotherapy was administered, and the patient remained clinically stable.

**Table 1:** Demographics, clinical features, and CSF biomarker results

	Age (years old)	Sex	Clinical syndrome	Onset of symptoms (days)	Aβ _40_ *(pg/ml)*	Aβ _42_ *(pg/ml)*	Aβ _42/_ Aβ _40_ Ratio	p-tau *(pg/ml)*	t-tau *(pg/ml)*
Case 1	45	M	Recurrent lobar hemorrhage	830	1,159.2	327.9	0.28	24.6	186.5
Case 2	73	F	Rapid cognitive decline	23	5,016	301	0.06	69.5	581
Case 3	71	M	Rapid cognitive and gait disturbance	70	4,419.0	368.84	0.08	27.2	656.2
Reference range				−	−	≥ 630	> 0.095	< 61	≤ 290

Abbreviations: F, female; M, male.

### Case 2

A 73-year-old woman presented with a 3-month history of rapidly progressive cognitive decline, including disorientation and executive dysfunction. On admission, her score on the Mini-Mental State Exam (MMSE) was 8/30. Brain MRI showed a right frontal hematoma, vasogenic edema, and multiple lobar microbleeds ( Figure 1 ). Differential diagnoses, including infectious, neoplastic, and autoimmune causes, were excluded. Probable CAA-ri was diagnosed based on clinicoradiological criteria. The patient received intravenous methylprednisolone (1 g/day for 5 days), with improvement in cognition (MMSE 21 at discharge). At the 9-month follow-up, she maintained partial functional recovery. Cerebrospinal fluid biomarkers, obtained later in outpatient follow-up, showed reduced Aβ40 and Aβ42 ( Table 1 ).

### Case 3

**Figure 2:**
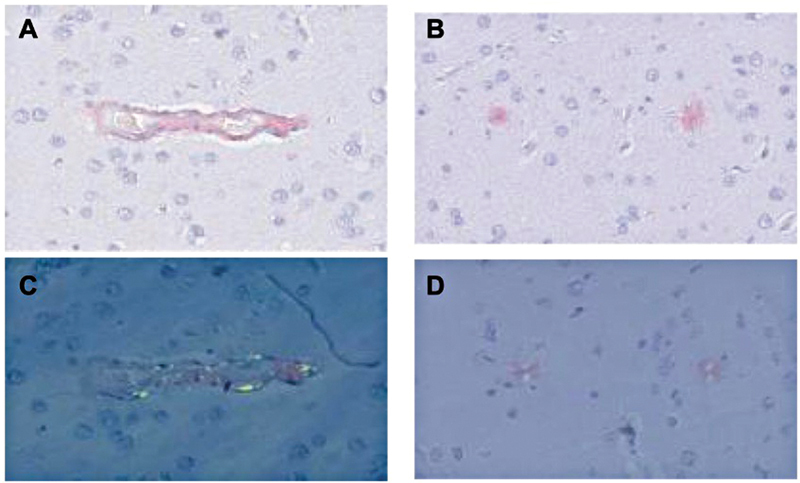
Brain biopsy from patient 2 (A) Congo red staining shows amyloid deposition in leptomeningeal and cortical vessels. (B) Immunohistochemistry with anti-Aβ confirms vascular amyloid positivity. (C) Bielschowsky silver stain highlights neuritic plaques. (D) Perivascular and parenchymal inflammatory infiltrates are visible around amyloid-laden vessels, consistent with a diagnosis of CAA-ri.

A 71-year-old previously healthy man developed gait ataxia, disorientation, incoherent speech, and visual hallucinations that progressed over 1 month to marked functional decline. Magnetic resonance imaging (MRI) demonstrated a large right temporo-occipital corticosubcortical lesion with vasogenic edema, lobar macrohemorrhages, and numerous microbleeds ( Figure 1 ). Cerebrospinal fluid showed lymphocytic pleocytosis (93 cells/mm ^3^ ), pronounced hyperproteinorrhachia (179 mg/dL), and hypoglycorrhachia (46 mg/dL), initially raising suspicion for tuberculous meningoencephalitis. After extensive microbiological studies remained negative and empirical antituberculous therapy yielded no improvement, a stereotactic biopsy was performed, confirming congophilic vascular amyloid with perivascular lymphocytic inflammation and hemorrhage ( Figure 2 ), establishing a definitive diagnosis of CAA-ri. High-dose intravenous methylprednisolone produced only transient cognitive improvement, and his prolonged hospitalization was complicated by nosocomial infection. Follow-up CSF biomarkers demonstrated reduced Aβ40 and Aβ42 concentrations ( Table 1 ).

The present retrospective case series included three patients diagnosed with CAA based on clinical and radiological findings at Hospital Geral de Fortaleza (HGF, in the Portuguese acronym), a tertiary public hospital in Fortaleza, state of Ceará, Brazil, between 2022 and 2024. Data collection involved neurological assessments, brain MRI with susceptibility-weighted imaging, and CSF analysis. Lumbar puncture was performed to investigate atypical presentations, such as rapidly progressive cognitive decline or suspected inflammatory syndromes. Cerebrospinal fluid biomarkers (Aβ40, Aβ42, t-tau, p-tau) were measured via enzyme-linked immunosorbent assay (ELISA) or chemiluminescence assays. The study was approved by the institutional Ethics Committee (approval number 76920623.2.0000.5040), with a waiver of informed consent. The present report follows the CARE guidelines (CARE) Act guidelines.

## FROM PRESENTATION TO RESOLUTION: LESSONS LEARNED

### What is CAA and which are its subtypes?

Cerebral amyloid angiopathy is a small vessel disease characterized by the deposition of β-amyloid in cortical and leptomeningeal vessels, resulting in various clinical manifestations, including lobar intracerebral hemorrhage and vascular cognitive decline. 1

Less common but clinically important types include CAA-ri and iatrogenic CAA (ICAA). Cerebral amyloid angiopathy-related inflammation is marked by an inflammatory reaction to vascular amyloid deposits, usually manifesting as subacute cognitive decline, behavioral changes, focal deficits, seizures, or headaches. 2 The 2016 criteria for probable CAA-ri require:

- age ≥ 40 years old;
- at least one characteristic clinical feature;
- asymmetric white matter hyperintensities on MRI extending to the subcortical regions;
- evidence of lobar microbleeds, cortical superficial siderosis or macrobleeds; and
- exclusion of other etiologies. 2

These criteria allow for noninvasive diagnosis with high specificity (97%) and good sensitivity (82%), helping avoid brain biopsy in selected cases. 2

Recent findings endorse CSF testing for anti-amyloid-β autoantibodies as a diagnostic method for CAA-ri, especially in atypical or nonhemorrhagic cases. 3 Updated meta-analyses indicate a distinct CSF biomarker profile in sporadic CAA, revealing decreased levels of Aβ42, Aβ40, and Aβ38, along with increased total tau and phosphorylated tau compared with healthy controls and Alzheimer's disease (AD) groups. 4 These profiles could improve diagnosis in unclear cases.

Iatrogenic CAA, on the other hand, refers to amyloid angiopathy resulting from previous exposure to cadaveric dura mater or neurosurgical instruments contaminated with Aβ seeds, particularly in individuals < 55 years old without a genetic predisposition. Proposed in 2022, the criteria for ICAA emphasize:

- clinical features compatible with CAA;
- absence of hereditary mutations;
- documented iatrogenic exposure;
- radiological findings consistent with CAA; and
- exclusion of alternative causes. 5

These criteria aim to standardize diagnosis and promote early recognition of this underdiagnosed form of Aβ-related pathology.

Integrating these diagnostic frameworks with updated neuroimaging criteria such as Boston 2.0 6 and CSF biomarker profiling enables a more comprehensive approach to identifying and managing rare CAA variants.

### What are the clinical spectrums of CAA?

Cerebral amyloid angiopathy encompasses a broad clinical spectrum, ranging from asymptomatic forms to lobar intracerebral hemorrhage, transient focal neurological episodes, cognitive impairment, and spontaneous cortical subarachnoid hemorrhage. These manifestations reflect progressive vascular amyloid deposition and its associated hemorrhagic and ischemic complications. 7 8

Among the less common but clinically significant variants is CAA-ri, an autoimmune encephalopathy caused by an immune response to vascular Aβ, likely mediated by antiamyloid β autoantibodies in the CSF. 3 Cerebral amyloid angiopathy-related inflammation typically presents with subacute cognitive decline, seizures, focal deficits, behavioral changes, or headache, in association with vasogenic edema on MRI.

### What are the current CAA and CAA-ri diagnosis criteria, and what challenges are associated with their application in clinical practice?

The in vivo diagnosis of sporadic CAA relies on the Boston Criteria 2.0, which integrate clinical data with MRI findings. These criteria incorporate both hemorrhagic markers (lobar microbleeds, cortical superficial siderosis) and nonhemorrhagic markers to enhance diagnostic accuracy. 6

For CAA-ri, validated clinicoradiological criteria allow for a noninvasive diagnosis with high specificity. Key features include the presence of characteristic clinical symptoms (e.g., subacute cognitive decline), evidence of hemorrhagic lesions typical of CAA on MRI, and extensive, asymmetric white matter hyperintensities representing vasogenic edema, after excluding other causes ( Table 2 ). 2

**Table 2:** Adaptable table based on the Criteria for the Diagnosis of Cerebral Amyloid Angiopathy-Related Inflammation, by Auriel et al. 2

Diagnosis	Criteria
Probable CAA-ri	**1. Age ≥ 40 years old** 2. The presence of at least one of the following clinical signs*: • Headache • Decrease in consciousness • Behavioral change • Focal neurological signs and seizures 3. MRI reveals unifocal or multifocal WMH lesions (corticosubcortical or deep) that are asymmetric and extend to the immediately subcortical white matter** **4. Presence of at least one of the following corticosubcortical hemorrhagic lesions:** • Cerebral microbleed • Cortical superficial siderosis **5. Absence of neoplastic, infectious, or other cause.**
Possible CAA-ri	**1. Age ≥ 40 years old** 2. The presence of at least one of the following clinical signs*: • Headache • Decrease in consciousness • Behavioral change • Focal neurological signs and seizures **3. MRI shows WMH lesions that extend to the immediately subcortical white matter** **4. Presence of ≥ 1 of the following corticosubcortical hemorrhagic lesions:** • Cerebral microbleed • Cortical superficial siderosis **5. Absence of neoplastic, infectious, or other cause.**

Abbreviations: MRI, magnetic resonance imaging; WMH, white matter hyperintensity.

Notes: *The presentation is not directly attributable to an acute intracranial hemorrhage (ICH); **The asymmetry is not due to past ICH.

Histopathology remains the reference standard method for definitive diagnosis, although clinicoradiological criteria are widely adopted in routine practice. 3

Despite their high specificity, the Boston Criteria were primarily developed and validated in cohorts with symptomatic lobar intracranial hemorrhage, representing a late stage of the disease. Consequently, their diagnostic sensitivity may be lower in patients without hemorrhagic presentations or in the earlier stages of CAA. This limitation highlights the need for supplementary diagnostic tools, such as molecular biomarkers, to improve the accuracy of noninvasive assessments, especially in atypical cases. 4 9

### How can CSF biomarkers aid in CAA diagnosis?

Analysis of CSF biomarkers offers a promising pathway to refine the differential diagnosis of CAA from AD. While CSF in both conditions shows reduced Aβ42 levels, this marker's utility for differentiation is limited due to its comparable levels between the two disorders (ratio of means [RoM] 0.88; *p* = 0.247). 4

In contrast, greater diagnostic specificity emerges from other Aβ isoforms. An updated meta-analysis including 289 CAA cases demonstrates that, compared with AD, CAA exhibits a more profound reduction in both Aβ40 (RoM 0.72; 95% confidence interval [CI]: 0.65–0.80) and Aβ38 (RoM 0.55; 95%CI: 0.38–0.81). Cohort data corroborate this, indicating that lower Aβ40 levels contribute to the distinction between CAA and AD (area under the receiver operating characteristic [AUROC]: 0.76). 10

Therefore, this biochemical signature—a pronounced reduction in Aβ40 and Aβ38 against a background of similarly low Aβ42—may provide a quantitative, noninvasive tool to substantially increase accuracy in the challenging differential diagnosis of CAA. 4 10

### What insights can be drawn from the reported cases and their clinical implications?

The present case series illustrate the practical application of a multimodal diagnostic approach and reports two distinct CAA phenotypes—one of ICAA and two of CAA-ri—underscoring the broad clinical spectrum of the disease and the value of a multimodal diagnostic approach. Our ICAA case (case 1), presenting with recurrent hemorrhages in early adulthood following a childhood neurosurgical intervention with dural grafting, aligns with emerging evidence of iatrogenic Aβ transmission. The diagnosis was supported by characteristic neuroimaging and a CSF profile of reduced Aβ40 and Aβ42. 5 11

The two CAA-ri cases (cases 2 and 3) demonstrated the characteristic subacute cognitive decline and MRI findings of vasogenic edema. Both cases responded to corticosteroid treatment, reinforcing the importance of early diagnosis and intervention in this treatable encephalopathy. The CSF findings of reduced Aβ40 and Aβ42 in both patients align with the expected biomarker profile for CAA and its variants. 4 Furthermore, the elevated p-tau in one case suggests that concomitant AD pathology may be present, a factor that could influence prognosis or long-term management.

These observations underscore the potential utility of CSF biomarker profiling—when interpreted in conjunction with clinical context and imaging findings—as an adjunctive tool in diagnosing atypical or early-stage CAA variants, potentially informing earlier therapeutic interventions. 4 12

Although limited by its retrospective, single-center design and lack of a control group, the present case series provides exploratory insights into the evaluation of rare CAA phenotypes. The findings support integrating CSF biomarker analysis with clinical and imaging data to enhance diagnostic confidence, particularly in complex or atypical presentations such as suspected CAA-ri and ICAA.
